# Butterfly wing architectures inspire sensor and energy applications

**DOI:** 10.1093/nsr/nwaa107

**Published:** 2020-05-23

**Authors:** Maurice I Osotsi, Wang Zhang, Imran Zada, Jiajun Gu, Qinglei Liu, Di Zhang

**Keywords:** butterfly wing architectures, bio-inspired, functional materials, sensors, energy applications

## Abstract

Natural biological systems are constantly developing efficient mechanisms to counter adverse effects of increasing human population and depleting energy resources. Their intelligent mechanisms are characterized by the ability to detect changes in the environment, store and evaluate information, and respond to external stimuli. Bio-inspired replication into man-made functional materials guarantees enhancement of characteristics and performance. Specifically, butterfly architectures have inspired the fabrication of sensor and energy materials by replicating their unique micro/nanostructures, light-trapping mechanisms and selective responses to external stimuli. These bio-inspired sensor and energy materials have shown improved performance in harnessing renewable energy, environmental remediation and health monitoring. Therefore, this review highlights recent progress reported on the classification of butterfly wing scale architectures and explores several bio-inspired sensor and energy applications.

## INTRODUCTION

Worldwide population increase is causing environmental degradation, health deterioration and depleting existent energy resources [1]. Hence, environmental conservation, health intervention measures and development of clean and renewable energy technologies including sunlight, wind and water wave energies, have drawn global interest [2]. To date, many technologies exist for environmental conservation, health management and renewable energy harnessing. However, most of these conventional technologies have shown the need for performance to be improved [3]. Therefore, research attention should focus on developing efficient and high-performance technologies.

For centuries, living organisms have been constantly evolving intelligent systems characterized by their ability to detect changes in the environment, store and evaluate information, and respond to external stimuli [4]. The intelligent systems show outstanding physiochemical properties and stimuli responsiveness, such as structural coloring of butterfly wings [5], structural color changing in chameleons [6] and thermal insulation in polar bears [7]. Specifically, butterfly wings exhibit architypes of unique micro/nanostructures, vivid wing coloration, light-trapping mechanisms and responses to various stimuli [8]. These naturally intricate features have been replicated into man-made functional materials for applications in sensors [9], photovoltaics [10], photocatalysis [11], biomedicine [12] and robotics [13]. However, existent technologies are incapable of fully replicating these natural intelligent systems into man-made functional materials [14]. Therefore, research is exploring effective replication techniques and performance enhancement of bio-inspired functional materials.

Bio-inspiration explores biostructures and their intelligent systems replication into man-made functional materials [15]. Since microscopy invention by Hooke in 1665, early butterfly wing research focused on investigating biological characteristics [16,17]. Researchers including H. Ghiradella, P. Vukusic and D. Stavenga pointed out that chitin (polysaccharide, [C_8_H_13_O_5_N]_n_) is the main building material in butterfly wing scales. They characterized wing scales to have fair periodicity, interchangeable high and low refractive indices and dimensions in the range of a few hundred nanometers [18–22]. Afterwards, chemistry and material science characteristics gained popularity. For example, Siddique *et al*. determined that iridescent coloration in *Hypolimnas salmacis* resulted from the lower lamina of cover scales as opposed to upper lamina architectures [23]. Likewise, many researchers have presented research findings on wing architectures and their bio-inspired sensor and energy applications. However, in-depth research is necessary to improve this knowledge and develop more efficient bio-inspired functional materials.

Currently, butterfly architectures research has progressed from micron to nanometer scale. The subtle architecture sensitivity and refractive index responsiveness are important properties in sensor applications. Similarly, energy-harnessing applications rely on wing properties including vivid wing coloration and light-trapping mechanisms [24,25]. Thus, diversity in butterfly species prompted previous classification of their architectures into three and seven categories, respectively [26,27]. Initially, Ghiradella explored the patterning of structures in butterfly scales [22]. Also, Vukusic *et al.* studied the structures of butterfly architectures and classified them into three categories (types I, II and III scales) [21,28]. Recently, our group balanced the simplicity and comprehensiveness of the previous groupings in a summarized five categories classification, based on specialized regions of butterfly wing scales [29]. In subsequent years, numerous studies on wing architectures have presented many findings. However, inclusion of recent findings to update our classification system is inexistent to the best of our knowledge.

Thus, this review systematically explores the structural appearance of butterfly wing scale architectures and distinguishes different architectures based on variations in their specialized regions. These wing architectures influence the properties and characteristics of the whole wing including the porous structure, large surface area, stimuli responsiveness and light manipulation. Subsequently, application of these wing properties in selected butterfly wing inspired sensor and energy systems is highlighted, while drawing a comparison with similar systems inspired by other biological species. The review also discusses various synthesis techniques used in fabricating butterfly wing inspired materials in the supporting information. Lastly, the review points out key areas of future research advancement.

## OVERVIEW OF BUTTERFLY WING SCALE ARCHITECTURES

### Butterfly wing scales

Lepidopteran insects are attracting immense research and aesthetic interest worldwide [30]. Lepidopteran research focuses on wing scales and the appearance of their vivid colors [31]. Their architectures interact with light by wavelength selective reflection or coherent scattering to produce structural coloration [32]. Equally, light interacts with pigments through wavelength selective absorption or reflection of incoherently scattered light to form pigment coloration [33–36]. Previously, coloration research explored ∼47-million-year-old lepidopteran moth fossils by reconstructing their structural coloration. The results showed a yellowish-green coloration on the dorsal surface of the forewings, functioning as a warning signal during feeding [37]. Recently, research explored the scale architectures of Jurassic lepidoptera [38]. These lepidopterans had one bilayer scale of fused cover scales in the upper layer and small fused ground scales in the lower layer. The wing architectures were characterized by uneven ordering, small sizes, ridge ornamentation and periodicities between 140 and 2000 nm, for visible light scattering. The fused wing scales and visible light scattering coloration are obvious earliest indications of an ancient lepidopteran evolution process. While lepidopteran insects comprise moths and butterflies, huge research attention has focused on butterfly wings. Nevertheless, other butterfly parts such as proboscis, have recently been applied in bio-inspired soft actuator applications [39]. Hereafter, the discussions will focus on the progress of butterfly wing architectures research.

Butterfly wings research seeks to understand the single wing scale cell development process, their specific shape, the unique cuticular architecture patterning their surface, and their influence on evolutionary, behavioral and ecological processes [40]. Generally, lepidopteran wing scale development begins at pupal stage. Each cell body is positioned inside the wing epithelium and generates a cytoplasm-filled extension. The extension is enclosed in an active cell membrane extending out of the epithelium [18,41–43]. Initially, extensions are cylindrically shaped but become flattened during the growth process to result in wing scales. Towards the end of the pupal stage, the cell dies and forms wing scale architectures of chitin filled with air and pigment [44].

**Figure 1.**
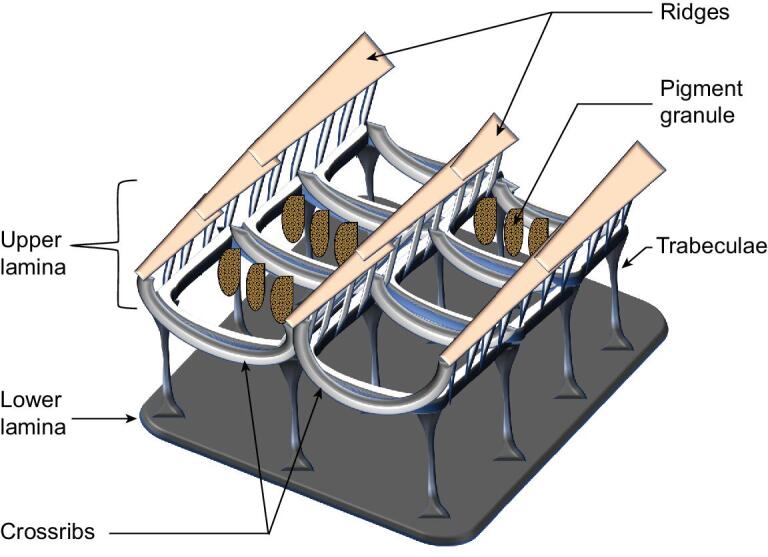
Schematic diagram showing features and architectures of a typical butterfly wing scale.

Further characterization shows that a typical wing scale resembles a flattened sac of about 200 μm length, 75 μm width and 5 μm thickness [45]. Each flattened sac has a visible and highly convoluted upper lamina, interiors and a plate-like and featureless lower lamina (Fig. 1) [46]. The upper lamina is a reticulate architecture of ridges aligned parallel to the longitudinal axis. The ridges are connected at intervals by arch-structured cross-ribs, positioned transverse to the longitudinal axis. In the scale interior, a hollow space (window) is formed between the ridges and cross-ribs. This upper lamina is connected to the plate-like lower lamina by pillar-like structures called trabeculae. Within the lumen of some butterfly wing scales, pigments except melanin are held in pigment granules, while melanin is distributed in the wing scale structures [27,29,47].

The wing usually comprises numerous scales organized in alternate rows of long and more specialized cover scales overlapping and concealing the shorter, less specialized ground scales. The cover and ground scales exhibit structural coloration by diffusely scattering and reflecting light, respectively [48]. However, some species of butterflies such as those in the genus *Greta*, have transparent wings for camouflage against predators [49]. The transparent areas of *Greta andromica* butterfly wing were previously investigated by bifurcated probe measurement. The results indicated a lack of wing scales on the transparent parts and yielded a reflectance spectrum having distinct oscillations, contrary to thin-film characteristics [50]. Similarly, research on the transparent butterfly wing of *Greta oto* (glasswing) revealed numerous randomly sized, high aspect ratio nanopillar (nipple array) structures, exhibiting omnidirectional anti-reflection characteristics [51]. These anti-reflective nipple arrays are also found in hawk moths and cicada wings [52,53].

**Figure 2.**
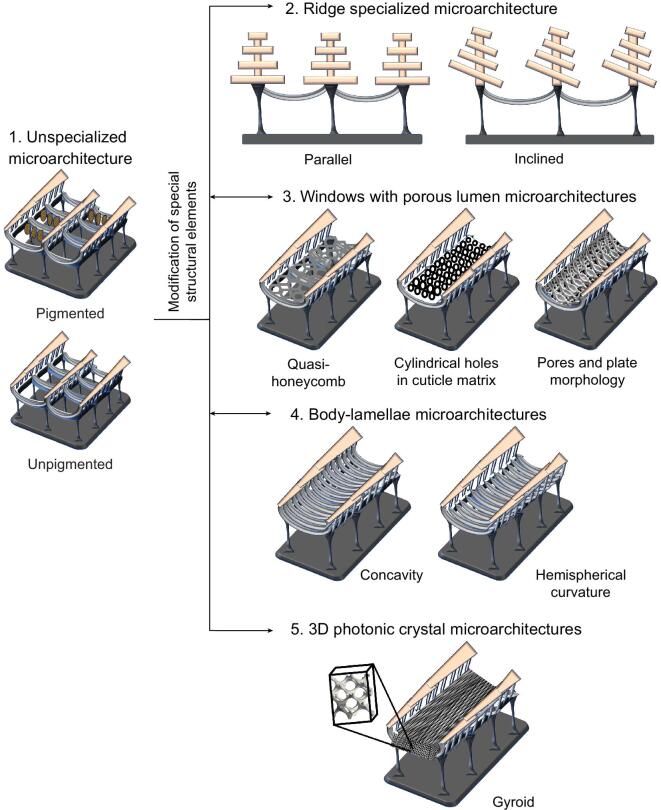
Classification of butterfly wing architectures based on the specialized regions of the wing scales [29].

In this section, we discuss the summarized five categories of butterfly wing microarchitectures, which have been distinguished according to variations in the specialized regions of the wing scale (Fig. 2) [29]. The first category comprises unspecialized microarchitectures, characterized by a convoluted upper lamina having longitudinal ridges with overlapping lamellae. These ridges are connected at intervals by transverse cross-ribs. Hollow spaces form between ridges and cross-ribs (windows) towards the wing scale interior. This interior may have pigment granules containing pigments [18]. The modification of special structural elements in the microarchitectures results in formation of the other categories including ridge specialized microarchitectures (parallel or inclined), windows with porous lumen microarchitectures (quasi-honeycomb, cylindrical holes in cuticle matrix or pores and plate), body-lamellae microarchitectures (concavity or hemispherical curvature), and 3D photonic crystal architectures (gyroid).

### Category 1: unspecialized microarchitectures (pigmented or unpigmented)

The general architecture of butterfly wing scales resembles a flattened sac consisting of visible and highly convoluted upper lamina, interiors and a plate-like featureless lower lamina. Dense pigmentation in the scale interior causes pigment coloration by wavelength selective absorption or reflection of incoherently scattered light [54]. Pigments account for a majority of the yellow, red, orange and black-brown color in butterflies [21]. The contrast of refractive index with adjacent air causes the peripheral portions of wing scales to show strong light scattering [36]. Some butterfly species with dark brown or black color have melanin pigments, which exhibit high light absorption across all visible wavelengths and heightened absorption in shorter wavelengths [55]. For example, *Pieris rapae* wing scales contain numerous ovoid pterin pigments, compacted in randomly ordered grain-shaped granules. Their ordering enhances the scattering strength and reflectivity of the wing scale, resulting in a strong color [56]. Conversely, the lower lamina determines the wing scale color in the absence of pigmentation [57]. The lower lamina in species such as *Eryphanis aesacus* and *Celastrina ladon* has a thickness in the order of 200 nm and functions like an optical thin film, causing specific wavelength-selective reflectance [45,58,59]. In addition, the unpigmented architecture resembles two crossing venetian blinds, where the periodic arrangement of ridges forms flat diffraction planes above similar but smoother cross-rib planes [29]. By definition, diffraction is the bending of light around an obstacle or aperture forming a geometric shadow [60].

### Category 2: ridge specialized microarchitectures (parallel or inclined)

In some species, the upper lamina has specialized ridge microarchitectures with overlapping periodic lamellae, exhibiting angle-dependent iridescent structural coloration. Thus, the wing scales change colors depending on the observation angle relative to the perpendicular axis [61]. The alignment angle of these multilayered interference microarchitectures relative to the lower lamina is either parallel or inclined [21]. In detail, parallel ridge specialized microarchitectures entail dendritic-like architectures resembling Christmas trees. Observed perpendicular to the axis of the ridge, the overlapping lamellae resemble tree branches attached to a stem (ridge) [62]. Spacing between the ridges and the number of lamellae influence the wing scale colors [63]. For instance, larger spacing between two ridges or fewer lamellae cause darker colors [64]. *Morpho didius* have periodic parallel ridge specialized microarchitectures of alternating chitin and air multilayered lamellae. The multilayered lamellae structure has unique light wavelength selective abilities, forming eye-catching metallic blue iridescent structural coloration [65]. Conversely, deviation from perpendicular alignment to the lower lamina develops an inclined ridge specialized microarchitecture having intra-ridge layers [66]. In small and moderate inclination angles, the solid angle of reflection is constrained. This results in a larger range of colors. The small and moderate inclination angles generate a strong on–off color flashing effect, even with minimal wing movement like in *Ancyluris meliboeus* [67]. Steeper inclination angles narrow the solid angle of reflection, resulting in limited iridescent coloration. For instance, steeper inclination angles induce dark and bright zones, only observable at grazing incidence from the rear of the species like *Troides magellanus* [68–70].

### Category 3: windows with porous lumen microarchitectures (quasi-honeycomb, cylindrical holes in cuticle matrix or pores and plate)

The upper lamina has hollow spaces (windows) between the ridges and the cross-ribs. Cross-ribs specialization causes the space under the window to have different porous patterns or hollow networks, trapping light within the architectures and creating a dark wing appearance [49]. Variations in these hollow networks produces the quasi-honeycomb architecture, cylindrical holes in the cuticle matrix and pores and plate morphology. Quasi-honeycomb architecture is characterized by a hollow structure resembling a disordered honeycomb. This architecture scatters or diffuses light within the structure and then reflects it off the wing scale cuticle. Light interaction with quasi-honeycomb architecture produces a darker wing scale appearance, such as in *Papilio paris* and *Papilio bianor ganesa* [71–73]. However, the upper lamina may generate a thick 2D lattice with disordered air holes called cylindrical holes in the cuticle matrix. This architecture variation resembles a slab with hollow air cylinders enclosed in chitinous cuticle. Most species having this architecture show structural coloration, except for *Papilio zalmoxis*, which has pigment coloration [74]. Similarly, specialization of the cross-ribs and micro-ribs may form pores and a plate morphology. This architecture forms a membranous and perforated dense lining of twin spindle-shaped structures, resembling a thick thread stitching in the window surface between the ridges. The pores and plate morphology is observed in the dorsal region of *Catopsilia pomona* wing [54].

### Category 4: body-lamellae microarchitectures (concavity or hemispherical curvature)

The highly convoluted upper lamina may have a high density of cross-ribs that fully cover and eliminate the spacing between the ridges. This architecture forms a stack of multilayered ridges with alternating chitin and air layers [45]. The entire multilayer architecture may contain a concavity or a slight hemispherical curvature, with layer spacing influencing the optical properties and structural coloration [75]. Comprehensively, concavity architectures have multilayer stack structures that are inclined at about 45 degrees, relative to the base of the scale. This inclination makes the opposite sides of the concavity perpendicular to each other [76]. When incident light is perpendicular to the base of the concavity architecture in certain species, yellow light directly reflects off the bottom of the multilayered structure. However, blue light is retro-reflected from one side of the inclined surface to the orthogonally opposite inclined surface of the concavity. The light then returns parallel to the initial direction of incident light, inducing a geometric polarization rotation [77]. The merging of the two reflected lights produces a green wing coloration on species like *Papilio palinurus* [78]. A variation of the concavity architecture is the shallower hemispherical curvature architecture, having an inclination of less than 30 degrees to the base of the scale. Retro-reflection of incident light is almost inexistent in this architecture. Therefore, the only observable color is iridescent violet/blue color in species like *Papilio ulysses* [79,80].

### Category 5: three-dimensional (3D) photonic crystal microarchitecture (gyroid architecture)

Lastly, the 3D photonic crystal (gyroid) architecture is an extremely connected triply periodic minimal surface (TPMS) structure, with the unit cell size in the visible light wavelength range [81]. This architecture has maze-like channels that are chiral and comprise equally opposite handedness of helix-like ordering, causing the net chirality geometry to have zero mean curvature everywhere [82–84]. Chirality is the geometric property by which the mirror image of an object cannot be changed by rotation or translation to match the original object [85]. The gyroid architecture contains two different network-like continuous sub-volumes showing circular dichroism. The bigger continuous sub-volume is filled with air, while the smaller one is occupied with cuticle [40]. The mechanism of gyroid architectures formation involves secluded facetted crystallites with a conspicuous size gradient, as opposed to polycrystalline space-filling organization reported in earlier research [86]. In addition, the gyroid architecture has domains showing preferential orientation along (001) axis, which is perpendicular to the surface of the scale and linked to the biological, illumination and coloration functions [87]. The green coloration in species such as *Callophrys rubi* originates from coherent scattering of light and pigments found in the internal minute domains [29].

In general, the hierarchical microarchitecture characteristics influence the properties of the entire wing including vivid wing coloration, light-trapping mechanisms and responses to a variety of stimuli [88]. In this review, the wing architecture properties such as response to temperature or mechanical forces have been applied in thermal sensors and medical sensors, respectively. Correspondingly, porous architectures permitting vapor diffusion (vapor sensors) or the optical irradiation of iridescent wing scales (anti-counterfeit security devices) have resulted in measurable color changes. To date, numerous research findings have highlighted the influence of microarchitectures on the properties of the entire wing. For example, an increment in ridge density caused increased UV reflection [64]. Equally, the V-shaped posture of butterfly wings increased solar concentration by 42.3% at optimal angle [89]. These properties have inspired development of photocatalysis, energy harvesting and energy storage applications. Subsequently, we discuss butterfly-wing-inspired sensor and energy applications.

## SENSORS INSPIRED BY BUTTERFLY WING ARCHITECTURES

Sensors are intelligent systems with dynamic and reversible responses to environmental conditions (stimuli). They operate by detecting changes from external environments, processing the condition in their internal systems and producing an output according to the stimuli [4,90]. The stimuli responsiveness of sensors enables them to process and quantitatively measure external conditions including temperature, humidity, pH, biomolecules and vapors [91]. However, existing traditional sensors exhibit limitations that impede their efficiency and accuracy. These include, sophisticated microfabrication methods, narrow test ranges, low spatial resolution, low selectivity and difficulty in controlling the microstructures [92]. Therefore, the development of sensors that overcome these limitations is of great importance.

Sensors inspired by butterfly wing architectures show selective optical responses to various stimuli, depending on their coloring and patterning [93]. Their advantages include, easy and direct fabrication from the butterfly wings, easy control of the microstructures, large surface area, faster and selective response behavior to external stimuli, light weight and environmental friendliness [91]. Bio-sensor designs attempt to replicate the fine, subtle nanoarchitectures of butterfly wings, which influences stimuli-responsive visual signals. Control of the microstructures enables dynamic tailoring of the bio-sensors to detect different stimuli with heightened sensitivity and effectiveness [94]. The hierarchical architectures may produce a discernable visible response to subtle variation in environmental temperature (thermal sensors) and mechanical forces (medical sensors). Equally, diffusion of vapor (vapor sensors) or optical illumination of iridescent wing scales (anti-counterfeit security devices) results in measurable color changes. These four sensor distinctions are discussed hereafter.

### Thermal sensors

Temperature is an important parameter, whose monitoring and regulation guarantees safety of the system [95]. Thermal sensor functioning involves a selected thermo-sensitive material absorbing and converting temperature into thermal energy, followed by modification of the sensor's physical characteristics with temperature change [96]. Conventionally, thermal sensors are classified into thermocouples, thermistors and resistance temperature detectors [91]. These thermal sensors have disadvantages including high cost, complexity of microfabrication methods and low spatial resolution [94]. Therefore, development of thermal sensors demonstrating stability, accuracy, easy fabrication, low cost and efficient functionality is necessary.

Naturally, fire beetles have infrared receptors in their metathoracic pit organs for thermal detection of fires [97]. Similarly, butterfly wing nanoarchitecture inspired sensors are capable of transforming external stimuli into visually perceived signals for quantitative measuring of different stimuli including temperature, pH and vapors [98,99]. The hierarchical architectures may produce a discernible visible response to subtle variation in environmental temperature by thermal expansion and changes in the refractive index [25,94]. For example, previous reports inferred that *Morpho* architectures exhibit visible optical responses to temperature, mainly due to thermal expansion and mildly from refractive index variations, on detection of infrared radiation [100]. Another report on the *Polyommatini* butterfly species demonstrated that enhanced spectral signal responses result from elevated shifting of the spectral position with lowering temperatures [101]. For effective butterfly wing scale inspired thermal sensor applications, a good balance between the light manipulation properties and the thermal expansion or shrinkage characteristics is essential for good visual signals [75]. To boost the temperature detection range of some bio-inspired thermal sensors, incorporation of smart polymers and semiconductors into butterfly wing templates improves their thermo-responsive capabilities [102]. In addition, butterfly wing scales demonstrate advantages including low cost, easy fabrication process and easy control of the microarchitectures.

**Figure 3.**
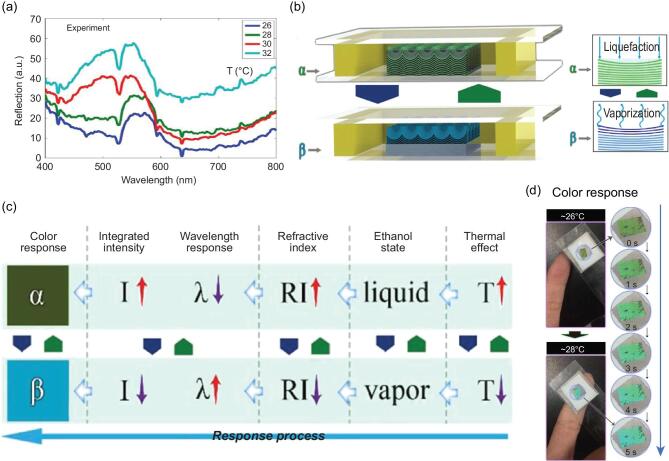
Butterfly-wing-inspired thermochromic sensor. (a) Thermochromic experiment: reflectance property of sealed PC wing sample at varied temperatures (26°C, 28°C, 30°C and 32°C). (b) Diagram of reversibility with α and β states (α, ethanol liquefaction; β, ethanol vaporization). (c) Response process: thermal effect, ethanol state, refractive index, wavelength and intensity response, and color response (red and purple arrows imply an increase or decrease of the parameter). (d) Color response from temperature variation due to the contact with a finger from 0 to 5 s. Republished with permission of [Walter de Gruyter and Company], from Ref. [103]; permission conveyed through Copyright Clearance Center, Inc.

Butterfly wing inspired thermal sensors rely on subtle wing scale architectures for the dynamic and reversible responses to temperature changes. Thermal expansion or shrinkage of architectures produces a color transformation detected as a

measurable visual signal [94,98]. Recently, *Papilio ulysses* butterfly wings were used in fabricating a thermochromic sensor comprising an ethanol-saturated wing sample. This was sealed and enclosed within two glasses and double-sided tape for reversibility (Fig. 3) [103]. The thermochromic sensor refractive index regulating mechanism was achieved by liquefaction and vaporization of volatile ethanol. Temperature increment volatized the ethanol and resulted in heightened peak value, wavelength variation and wing color changes. Therefore, thermal energy was converted to a visual color response signal via reversible response to light wavelength and intensity. This bio-inspired thermochromic sensor mechanism has the potential to be applied in thermal detection, photonic switches and displays.

Similarly, high-temperature bio-sensors require inclusion of special polymers having high performance and good thermal stability properties [104]. *Papilio paris* butterfly wing scales were recently used as bio-templates in an NIR-thermal sensor, comprising photothermal Fe_3_O_4_ and thermo-responsive poly(N-isopropylacrylamide) or PNIPAM coatings [98]. Refractive index manipulation using special polymer coatings enhanced the spectral performance of the butterfly wing scale architectures. When irradiated by NIR light, photothermal Fe_3_O_4_ nanoparticles rapidly converted NIR radiation into heat and initiated a phase transition of PNIPAM within 10 seconds. This phase transition caused a composite refractive index variation by a wavelength spectrum shift of about 26 nm, with obvious color changes for visible readout. Therefore, the durability, stability and detection mechanism of the NIR-thermal sensor resulted from the strong chemical bonding between PNIPAM and the butterfly wing template.

In principle, thermal sensors operate by the temperature-induced transformation of physical characteristics in thermo-sensitive materials upon heating or cooling [96]. Naturally, plant chlorophyll gives leaves their green color during spring but catabolizes into red or brown color under extreme summer and winter temperatures. Recently, a precursor molecular sensor (PMS) inspired by chlorophyll metabolism exhibited a color change signal at 180°C [105]. Incorporating the PMS into an early flame warning system resulted in a rapid colorful alarm at 275°C within 20 seconds. Thus, nature continuously inspires functional materials with improved performance from its huge catalogue of evolved biological species. This existent knowledge should propel further advancement and improvement of thermal sensor performance to match those found in natural biological species.

### Medical sensors

The human body contains a complex network of sensory systems for sight, sound, taste, smell and feel. Illnesses that affect these sensory systems often cause discomfort and/or severe impairment of these senses [106]. For example, neurodegenerative (ND) diseases such as Alzheimer's disease and Parkinson's disease, are chronic and progressive illnesses targeting extermination of neurons, especially in elderly people [107]. Similarly, glaucoma causes optical nerve damage by exerting increased intraocular pressure (IOP), resulting in irreversible blindness [108]. To manage these diseases, medical sensors are fabricated for diagnosis, therapy and health monitoring by measuring physical signals. However, traditional medical sensors such as electrograms use adhesion tape, straps or piercing needles to attach electrodes onto the organs or skin. In addition, decoding the received electrical signals is only possible through connection of electrodes to bulky and rigid computing devices [109]. For effective disease diagnosis and health monitoring, it is essential to develop flexible, ultrathin, simple mechanism and lightweight medical sensors compatible with the skin or organ attachment surface.

Efficient medical sensors for diagnosis and health monitoring must exhibit distinct advantages including flexibility, simplified mechanism, light weight, easy fabrication, compatibility with the attachment surface and low cost [110]. Naturally evolved systems have exceptionally intelligent mechanisms, which respond effectively to the external stimuli [90]. Currently, medical sensors incorporate functional materials on flexible supports for precise and sensitive detection of target indicators in lengthy health monitoring. Similarly, detection of biomarkers in body fluids has been achieved by integration of electronic and microfluidic systems [111]. Therefore, medical sensor systems utilizing natural architectures exhibit high sensitivity, light weight, compatibility with the attachment surface and accurate measurement of the indicators being monitored.

**Figure 4.**
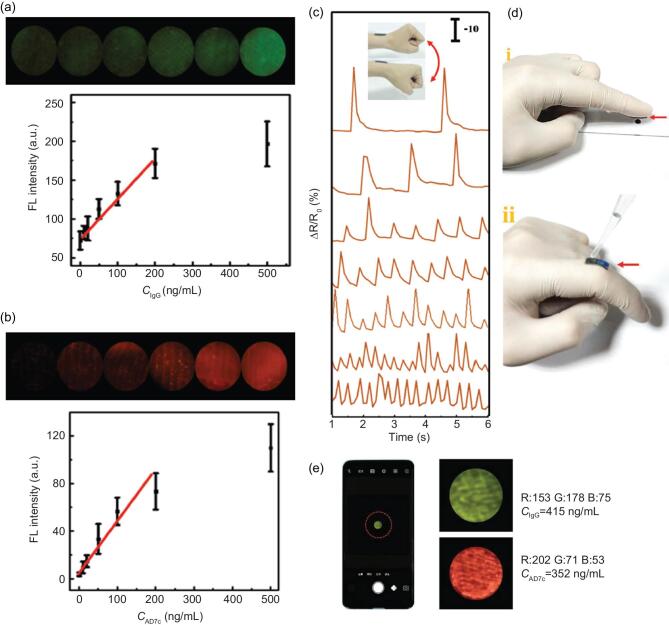
Integrated wearable medical sensor. (a) Fluorescence intensity of the detection reservoir in the chip as a function of the human IgG concentration. (b) Fluorescence intensity of the detection reservoir in the chip as a function of the AD7c-NTP concentration. (c) Relative resistance variations on wrist bending at different frequencies (relative change = 10%). (d) Photograph of the integrated wearable system attached on fingertip (i) and knuckle (ii) for body fluid sampling. (e) The fluorescence detection of IgG and AD7c-NTP with a smartphone-based device. Used with permission from Ref. [107]. Copyright (2018) Wiley-VCH.

ND diseases have complex causes without any identifiable cure. Hence, early diagnosis by identification of the biochemical markers, behavioral monitoring and treatment are the only intervention measures slowing its quick progress. Recently, *Morpho menelaus* butterfly wings inspired fabrication of a wearable medical bio-sensor (Fig. 4) [107]. The sensor design comprised an integrated microfluidic system and an electronic network, on the upper and lower layers of the wing, respectively. In detail, the integrated microfluidic system comprised a heterojunction of the wing upper layer and SiO_2_ nanoparticles. The microfluidic system heterostructure boosted the fluorescent intensity of two identifier proteins (AD7c-NTP and IgG), for multiplex detection using a smartphone-based device. The lower layer of the wing had an electronic network of conductive ink for physiological monitoring. This measured the rate of change in resistance and obtained static tremor frequencies from the ND disease patients. Fusion of the microfluidic chip and the electronic network using a double-sided tape achieved the mechanism of biochemical and physiological fluorescent signal detection in fluorescence immunoassay. The bio-sensor detected resistance variations from wrist bending over varied frequencies. Therefore, the wearable medical biosensor presents a hybrid system for physiological-biochemical monitoring of ND disease, with practical applications in human–machine interaction.

Similarly, glaucoma causes irreversible blindness by exerting increased IOP and damaging the optic nerve [108]. The recent development of a bio-photonic IOP sensor medical implant was inspired by architectures in the transparent parts of *Chorinea faunus* butterfly wings [112]. The sensor membrane was a composite of phase separated and immiscible polystyrene and poly(methyl methacrylate) polymer mixture on Si_3_N_4_ substrate. The bio-photonic nanostructured membrane showed an expanded optical readout angle, strong hydrophobicity, reduced swelling and antiseptic properties inhibiting bacteria adhesion. The IOP sensors with optomechanical sensing elements were implanted in the anterior chambers of New Zealand white rabbits for a month.

The monitoring of the eye with the implant showed no signs of inflammation. Thus, the sensor provides a practical implant for long-term IOP monitoring in glaucoma patients.

Another recent study compared fabricated poly(lactic acid) gyroid scaffolds with a spring shape, good porosity and mechanical properties, versus strut-based architectures in tissue engineering [113]. Their porosity and mechanical properties were measured by micro-CT and compression testing, respectively. The effect of geometry and crystallinity on the degradation of these architectures was measured *in vitro*. Consequently, the gyroid architectures demonstrated superior qualities with 71% porosity. Compression tests indicated isotropic behavior, a variation from the strut-based architectures. When they were aged under physiological conditions, gyroid scaffolds maintained their integrity for 64 weeks while control strut-based scaffolds started to lose struts from 33 weeks. The resilience of gyroid architectures was attributed to the printing resolution and their crystallinity. Therefore, gyroid architectures were proposed as the preferred mesh architecture for production of cheap and personalized implants in tissue engineering.

While medical diagnosis and health monitoring are the known remedies for incurable diseases, recent developments have emphasized the ability of medical sensors to monitor human motion and the biochemical markers simultaneously. Thus, the next generation of medical sensors must exhibit compatibility with the attachment surface, flexibility, light weight, easy fabrication, simple mechanism, quick response and low cost [110]. For example, recent research developed a wearable optical health sensor inspired by the radiant peacock tail feathers for diabetes disease management [114]. The sensor showed high sensitivity in electrochemical detection of lactoferrin protein in tears. Despite medical sensor advancements, the increasing number of incurable diseases demands performance and functional enhancement in developing early diagnosis and health-monitoring systems. Therefore, development of bio-inspired medical sensors should continuously focus on improving health conditions and increasing efficiency in monitoring diseases.

### Vapor sensors

Toxic and volatile organic compounds (VOCs) include hazardous gases, vapors and volatile liquid mediums, which pose a danger to the environment and human health if leakage occurs [115]. Specifically, the vapor sensing process entails diffusion of vapor into the porous microarchitectures and the surface reaction between targeted vapor and the absorbed oxygen on the semiconductor oxide surface. Material compositions and architectures influence vapor sensor efficiency, with open hierarchical porous architectures exhibiting higher amounts of transmission channels, for rapid vapor diffusion and more activated surface area with numerous reactive sites [116]. However, conventional vapor sensors are comprised of thick films, which decreases the diffusion rate [117]. Equally, single outputs of light intensity, resistance or capacitance detection have decreased sensor performance in multiple and complex stimuli environments requiring selectivity [118]. Therefore, development of sensor systems with porous structures, high surface area, excellent selectivity and fast response time is necessary.

Biological architectures have numerous macropores, permitting quicker molecular diffusion and vapor transport [119]. Butterfly wings have good porosity, structural coloration, refractive index responsiveness, rapid response times, unique thin-walled hierarchical architectures and a large surface area for faster diffusion and reaction of vapor [76]. Their sensing mechanism involves a color transformation on increasing vapor concentration and reverting back to the original color upon regaining an ambient environment [120]. Interaction of butterfly wing inspired sensors with various vapor concentrations produces different changes in the reflectance spectra, indicating the selective response characteristics of the architectures to particular vapors [121,122]. Therefore, butterfly wing scale architectures exhibit properties ideally suited for vapor sensing applications.

**Figure 5.**
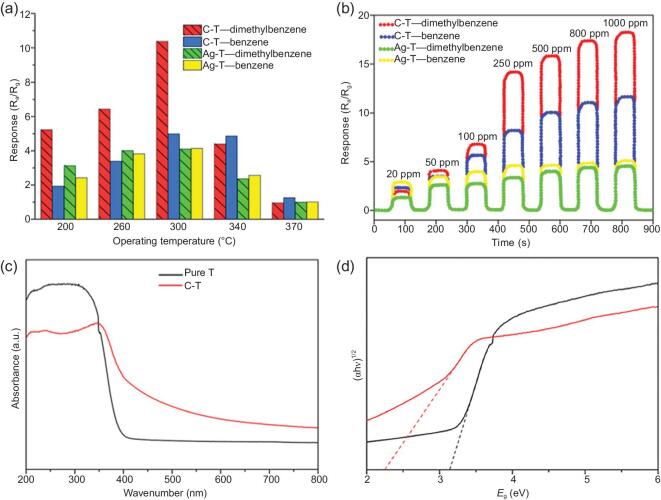
Biomorphic C/TiO_2_ vapor sensor. (a) Vapor responsiveness of C-T sensors and Ag-T sensors, respectively, in relation to the temperature at vapor concentration of 100 ppm and relative humidity of 10%. (b) Instantaneous sensing responsiveness of C-T and Ag-T sensors to benzene and dimethylbenzene in varied concentrations at operating temperature at 300°C and relative humidity of 10%. (c) UV-vis absorption spectra of pristine T and CT, and (d) Tauc plot showing evaluation of optical band gap of pristine T and CT. Reprinted with permission from Ref. [123]. Copyright (2018) American Chemical Society.

Butterfly wing porous hierarchical architectures exhibit an enhanced rate of vapor diffusion and color transformation for measurable detection [76,117]. Recently, *Papilio paris* architectures were applied as bio-templates in a biomass carbon-doped TiO_2_ (C/TiO_2_) vapor sensor (Fig. 5) [123]. On exposure to benzene and dimethylbenzene vapors at 300°C, biomorphic C/TiO_2_ exhibited high specific area (85.27 m^2^.g^−1^), excellent visible light sensitivity and excellent vapor responsiveness. The vapor sensor applied as a safe concentration indicator detected vapor response measurements and determined vapor concentrations with distinct visual signals. These excellent responses are attributed to the porous quasi-honeycomb architecture permitting vapor diffusion and the small grain sizes of TiO_2_ nanoparticles.

Likewise, chemical warfare agents (CWA) are lethal weapons with devastating consequences when leaked or detonated [124]. Traditional CWA detection methods including calorimetric, porous silicon and chemiluminescence are unsuitable for lengthy use. This is due to poor sensitivity, inadequate selectivity, sensor poisoning, expensiveness and portability challenges. Thus, ideal CWA sensor attributes include reliability, reusability, well-defined high analyte responsiveness, selectivity, rapid responses, durability and low cost [125]. Recently, *Morpho didius* wings were applied in the sensing of dimethyl methylphosphonate (simulated nerve agent) and dichloropentane (simulated mustard vapor) analyte vapors [126,127]. The periodic architectures of *Morpho didius* butterfly reflected the visible light and enabled reflectance measuring of each analyte in parts per million. To enhance CWA efficiency, alteration of periodic spacing within the lamella improved the spectral differences, while targeted nanostructure functionalization with binding materials improved target CWA binding. Therefore, the model presents a blueprint for designing selective, sensitive, quick-acting and cost-effective CWA sensors.

Undoubtedly, the porous hierarchical architectures increase the surface area and improve the molecular diffusion and vapor transport of the sensors [91]. In addition, bio-inspired vapor sensors assimilate biological systems advantages including light trapping, color transformation in vapor environment, thinness and refractive index responsiveness [128,129]. For example, the *Morpho* species demonstrates a color transformation based on a surface polarity gradient on the axis of the ridges, from the polarized upper part to the less polarized lower section [130–132]. However, the effect of inherent chemical composition, the complex biological applications and topographical characteristics on sensor functions have not been studied conclusively [93]. Therefore, investment in further research might propel improvement of vapor-sensor responsiveness and efficiency.

### Anti-counterfeit security devices

Forgery of important documents and currencies poses dangerous security threats. In principle, anti-counterfeit systems should be overt for visual perception, semi-covert for machine detection and covert for specialized equipment forensic detection [133]. Thus, the prime objective entails development of unique, complex and customized measures that are impossible to replicate and reverse engineer. To date, holograms are the dominant technique in anti-counterfeit applications [134]. Unfortunately, advanced printing techniques can access and reproduce the anti-counterfeit marks of holographic patterning [135]. Therefore, development of anti-counterfeit techniques that are unforgeable is of critical importance.

Naturally, butterfly wing structural colors exhibit outstanding optical properties that can be tuned to be resistant to fading, while maintaining excellent stimuli responsiveness to electrical fields, magnetic fields, chemicals, temperature, pH and mechanical forces [136,137]. Butterfly wing architectures exhibit distinctive optical properties, durability, uniqueness and easy visual perception in ambient environments. Their unique irregularities stem from disruptions in ridge array ordering, caused by variations in exact shapes between ridges and random spatial location deviations, such as ridge height. Hence, finding an exact wing scale duplicate is practically impossible, even for nature. This cell-level randomness results from non-deterministic relations between the genotype and phenotype [41,138]. Therefore, wing scale patterning and coloration exhibit dominant attributes for the next-generation anti-counterfeit applications.

**Figure 6.**
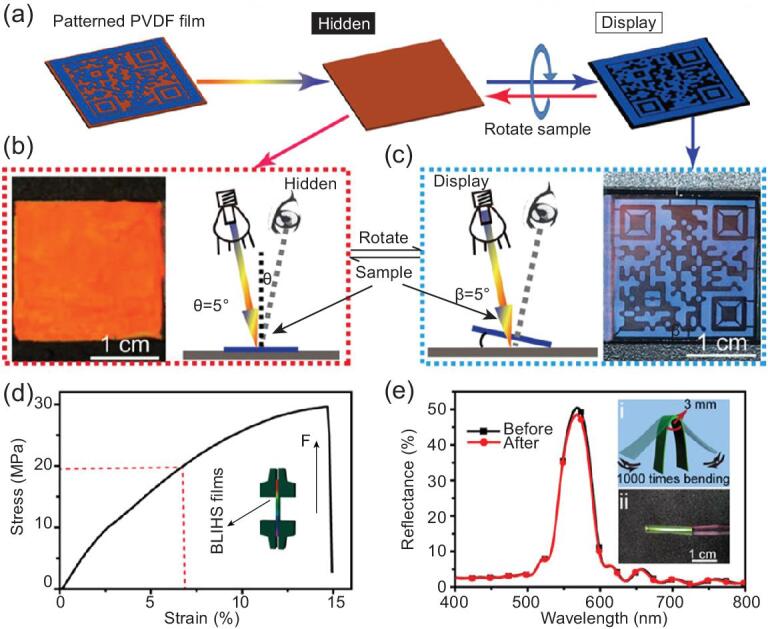
Bio-inspired PVDF film for anti-counterfeiting applications. (a) Patterned 320/240 BLIHS film displaying and hiding the QR code under different viewing modes. (b) Specular reflection mode diagram and red digital photo of the BLIHS film at the incident angles θ of 5°. (c) Diffuse reflection mode diagram and blue digital photo exhibiting the QR code pattern at β of 5°. (d) The tensile curve of the BLIHS PVDF films (29.7 MPa) and schematic of the tensile test (inset). (e) Reflectance spectra of the BLIHS PVDF film before and after 1000 times bending test; inset: (i) schematic diagram of the bending test and (ii) digital photos of the bent BLIHS PVDF film. Reprinted with permission from Ref. [140]. Copyright (2018) American Chemical Society.

Previously, multilayered architectures of *Suneve coronata* butterfly could be adopted and deposited as anti-counterfeiting security patterns on banknotes [139]. Recently, *Morpho* butterflies inspired fabrication of a polyvinylidene fluoride (PVDF) film for anti-counterfeiting applications using the templating technique (Fig. 6) [140]. The PVDF film with bilayer inverse heterostructure (BLIHS) consisted of two sets of architectures, namely, ordered array layers inverse architecture (OALIS) and quasi-amorphous array layers inverse architecture (Q-AALIS). Functionally, OALIS selectively displayed iridescent color according to Bragg's diffraction. Q-AALIS influenced the change to non-iridescent colors on rotating the film. This enabled the printing of intricate patterns (QR codes) on the PVDF film. Light scattering by Q-AALIS highlighted the non-iridescent color and eased the QR code scanning by a smartphone. The PVDF film showed outstanding inherent mechanical strength (29.7 MPa) and flexibility (1000 bending tests). Therefore, the bio-inspired PVDF film exhibited durability, uniqueness and ease of perception by an electronic device.

Evidently, replication of bio-structures in anti-counterfeit systems entails reproduction of the structure or its optical effect applied by a simpler structure [133]. The irregularity and random deviations in spatial locations makes butterfly wing architectures difficult to replicate. Hence, *Issoria lathonia* butterfly wings were applied as write-only memory security features [141]. Femtosecond laser-processing altered the wing surface and bleached it to imprint the digital signature of the authenticated document owner. This processing technique had numerous advantages including simplicity of the technique, inexpensiveness, easy production in large quantities and easy application to the existing anti-counterfeit security setup. The resultant anti-counterfeit security tag was durable and could not be replaced by any selected random butterfly wing.

Previously, numerous anti-counterfeit techniques were explored including nanotags, which generated sparsely random grown nanofibers as unique prints on a product. The prints could be imaged through low-magnification microscopy and digitally stored in a database for multiple uses [135]. Similarly, taggants design generated microparticles with self-produced wrinkle patterns resembling the human fingerprints, for passports and jewelry protection [142]. Despite their advantages, the intricate methodology used in developing these security systems makes natural systems the preferred choice. Irregularity of butterfly wing architectures showcases works of nature in distinguishing each structure, making them suitable for anti-counterfeit applications.

Generally, a bio-inspired sensing mechanism entails detection of an external stimuli, followed by a color transformation that can be quantitatively analyzed [94,120]. Bio-sensors exhibit many advantages including high sensitivity, easy and direct fabrication from butterfly wings, easy control of the microstructures, faster and selective response behavior to external stimuli and easy disposal due to eco-friendliness [91]. Among the numerous characteristics, the response of the wing architectures to temperature variations and mechanical forces, makes them suitable for thermal sensor and medical sensor applications, respectively. Similarly, numerous macropores in bio-inspired vapor sensors allow for quicker diffusion of vapors and surface reaction within the architecture. The vivid iridescent coloration and irregularities in wing architectures are important features in anti-counterfeit security applications. Therefore, butterfly wing inspired sensors exhibit good porosity, slenderness, structural coloration, refractive index responsiveness, easy fabrication and ecological friendliness.

## ENERGY APPLICATIONS INSPIRED BY BUTTERFLY WINGS

Statistical global energy production capacity is insufficient, propelling current attention to shift to energy production from abundantly available, greener and renewable energy resources [143]. For example, solar energy is an abundant renewable energy source with a light spectrum of ultraviolet rays (λ < 400 nm, 4%), visible light (400 < λ < 800 nm, 53%) and infrared rays (λ > 800 nm, 43%) [144]. Similarly, water covers 75% of the earth surface, with 97.5% of this constituting ocean and sea waters. This provides a huge potential source for harnessing tidal and water wave energy [145]. To date, numerous technologies have proven inefficient in the harnessing of renewable energies. Therefore, research continues to explore ways of boosting the energy-harnessing performance and improving the conversion efficiencies of renewable energy technologies.

Obviously, butterfly wings exhibit numerous properties including porous architectures, large surface area, stimuli responsiveness and light manipulation [146]. The porous architectures permit penetration of semiconductors into the gaps for easy replication. Also, their large surface area has numerous active sites for rapid charge transfer kinetics [147,148]. Hence, butterfly wing inspired energy materials exhibit easy fabrication, low cost, ecological friendliness and good stimuli responsiveness [8]. The bio-inspired energy materials have been applied in environmental remediation (photocatalysis), energy harvesting and energy storage systems, discussed hereafter.

### Photocatalysis applications

Photocatalysis is a process whereby catalysts absorb photons and generate energized electrons and holes, initiating reductive and/or oxidative (redox) reactions [149]. Photocatalysis demonstrates superior advantages over other techniques. They include ecological friendliness, capability of performance at ambient temperatures and efficiency to effectively degrade pollutants even at low concentrations [150]. The principal aim of photocatalysis is centered on the development of efficient photocatalysts that effectively utilize sunlight [151]. However, existent photocatalysts face numerous efficiency challenges including electron-hole pair recombination and huge band gaps (>3.0 eV) [152]. Therefore, research focuses on developing photocatalysts with improved performance and efficient solar energy utilization.

As a natural remedy, butterfly wing scale nanoarchitectures are easy to fabricate and control for enhanced performance of bio-inspired photocatalytic systems [153]. Their porous architectures allow the penetration of semiconductors into the gaps for easy replication into bio-inspired photocatalysts. Equally, the architectures have many active sites due to the large surface area for rapid charge transfer kinetics [148,154]. Therefore, current research focuses on increasing the photocatalyst surface area and enhancing photocatalytic efficiency through inhibition of electron-hole pair recombination and improvement of photoactivity [147].

**Figure 7.**
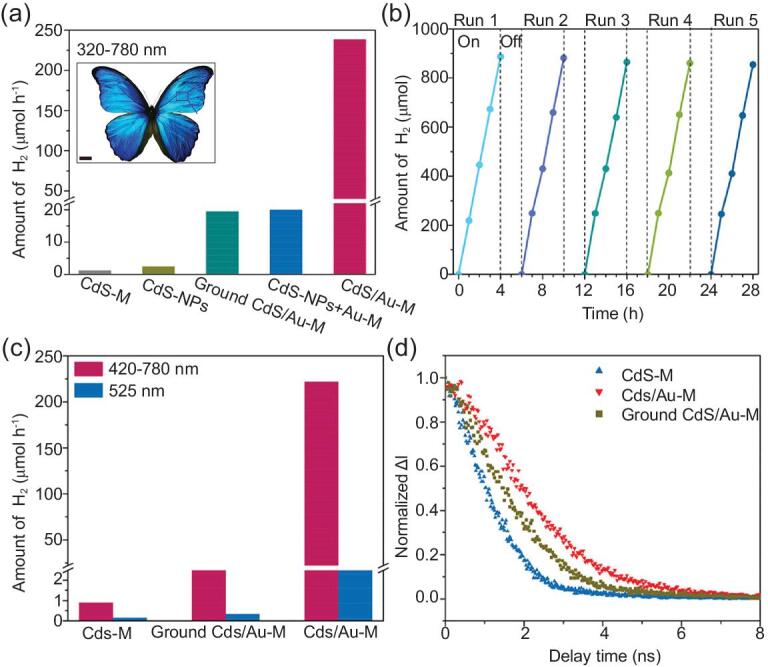
Photocatalytic hydrogen vapor production by bio-inspired 3D CdS/Au. (a) Photocatalytic activities of different samples under 320−780 nm incident light for H_2_ production (inset: photo of *Morpho didius* butterfly). (b) Cyclic stability of CdS/Au-M. (c) Photocatalytic activities of CdS-M, ground CdS/Au-M and CdS/Au-M under 420−780 and 525 nm incident light for H_2_ production. (d) Normalized time-resolved photoluminescence spectra of CdS-M, ground CdS/Au-M and CdS/Au-M, respectively. Reprinted with permission from Ref. [156]. Copyright (2018) American Chemical Society.

Normally, plasmonic metal photocatalysts such as Au, Ag and Pt exhibit surface plasmonic resonance by generating powerful electric fields upon strong light interaction, with restricted oscillations of free charged electrons on their surface [155]. Recently, *Morpho didius* wings inspired the fabrication of a bio-inspired 3D CdS/Au photocatalyst for hydrogen gas production (Fig. 7) [156]. Hydrogen gas is an eco-friendly and sustainable alternative fuel in industrial processes. However, it has low flammability, high volatility and high explosiveness [120]. For safety, the photocatalytic gas production was performed in a photoreactor with a closed gas circulation system and irradiated by artificial solar light at room temperature. The bio-inspired 3D CdS/Au plasmonic photocatalysis yielded a high hydrogen gas production rate of 221.8 μmol h^−1^ (241 times higher than the CdS butterfly photocatalyst) and good cyclic stability. The heightened productivity is ascribed to surface plasmonic resonance on multilayered butterfly architecture fused with Au metal and the impressive interfacial bonding states between Au and Cds nanoparticles.

Similarly, hierarchical gyroid architectures were printed using polylactic acid and implanted with TiO_2_ nanoparticles by a fused deposition modeling technique [157]. The resultant photocatalyst was used to degrade methylene blue dye under UV light illumination at a wavelength range of 280–360 nm. Light degradation on the surface of the polylactic acid allows the entrenched TiO_2_ nanoparticles to be constantly exposed on the hierarchical gyroid architecture surface. Therefore, the gyroid architecture photocatalyst maintained the reaction efficiency, had better adsorption and exhibited increased photocatalytic potential by forming more voids over several cycles. The bio-inspired photocatalyst has potential applications in water purification filters, energy-harvesting devices and drug delivery systems.

Clearly, photoactivity of any efficient photocatalyst is dominantly influenced by morphology, size and architecture [3,151]. For example, cicada wing architectures have inspired the fabrication of a biomorphic Ag-TiO_2_ photocatalyst for the degradation of methylene blue dye in 15 minutes. The exhibited high photoactivity was based on strong surface plasmonic resonance linked with biomorphic TiO_2_ [158]. Inclusion of nature-inspired hierarchical nanostructures has enhanced light absorption, increased the surface area for charge transfer kinetics and reduced the transportation lengths [153]. In addition, porous architectures permit the metal or semiconductor precursor to penetrate into the small gaps as opposed to depositing on the surface of the scale. This favors duplication of the fine periodic hierarchical architectures of butterfly wing scales [147,159]. Therefore, diverse butterfly wing specialized nano/microarchitectures present great potential for development of high-performance photocatalysts with heightened photoactivity.

### Energy-harvesting applications

Energy-harvesting technologies are mechanisms with huge potential for harnessing renewable energy [160]. Currently, three technologies of renewable energy harvesting have been developed, namely nanogenerators, biofuel cells and photovoltaic cells. In detail, nanogenerators are propelled by mechanical sources converting mechanical energy into electrical energy. However, their output power needs enhancement and storage. Biofuel cells convert chemical energy into electrical energy. Their performance is constrained by types and concentrations of biomolecules in the application [161]. Photovoltaic cells convert solar energy into electrical energy. Their performance is linked to the surface area of nanomaterials used such as TiO_2_, with a higher surface area enhancing solar light absorption from adsorbed dye [11]. Collectively, renewable energy-harvesting performance is hindered by low energy conversion efficiencies, resulting from low quantum conversion outputs and low optical absorption coefficients [162]. Therefore, improvement of conversion efficiencies is the key to elevated renewable energy-harnessing performance.

**Figure 8.**
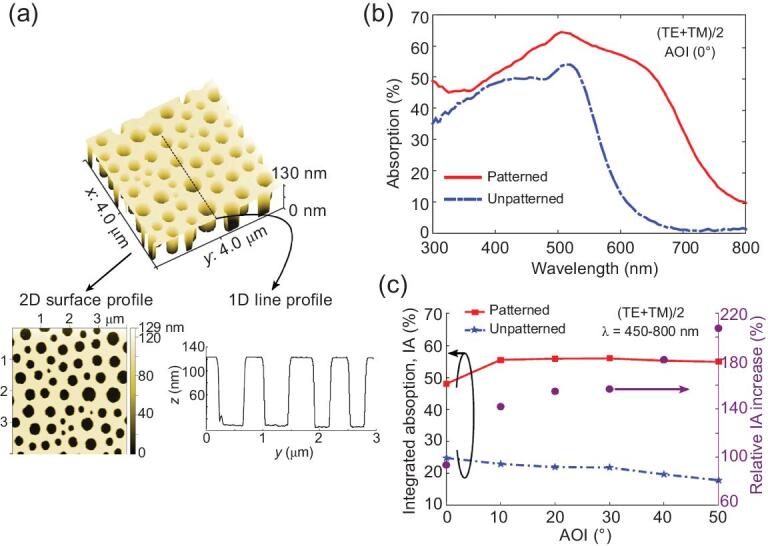
Bio-inspired patterned thin PV absorbers. (a) 3D AFM image of the bio-inspired a-Si:H thin film. The 2D surface profile indicates short-range ordered hole distribution for bio-inspired solar cell simulation. The 1D line profile shows uniform surface patterning with height (etching) profile. (b) The impact of the etched disordered nanoholes on the absorption spectrum is measured at normal AOI with unpolarized light. (c) Angular dependence of the IA for the patterned and unpatterned samples. Adapted from Ref. [164].

Currently, bio-photosynthetic architectures are inspiring efficient and cost-effective energy-harvesting photovoltaic materials. The materials exhibit increased solar light absorption, flexibility and increased surface area [163]. On the contrary, existing thin-film solar cells are exhibiting low efficiencies due to increased optical losses. Thus, the black butterfly wings (*Pachliopta aristolochiae*) inspired development of highly absorbing photovoltaic active layers (Fig. 8) [164]. The wing architectures had nanoholes for higher efficiency light absorption over a wider spectrum than smooth surfaces. Bio-inspired photovoltaic active layers comprised a blend of poly(methyl methacrylate) and polystyrene, mixed in methyl ethyl ketone, on a thin a-Si:H layer deposited on a glass substrate. The nano-patterned photovoltaic absorbers attained 90% increase in integrated light absorption at normal incident angle and up to 200% increment at large incident angles. These photovoltaic absorbers have potential applications in thin-film solar cells and solar light harvesting.

**Figure 9.**
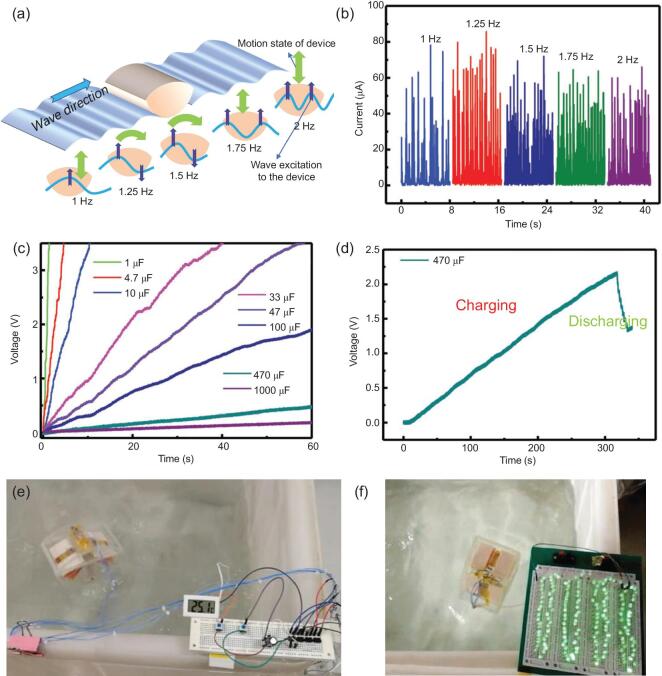
Butterfly-inspired triboelectric nanogenerator (B-TENG) for harnessing water wave energy. (a) Schematic diagram of B-TENG with the flat surface parallel to the incident wave of water. (b) The corrected output current of the B-TENG at several frequencies of water waves when the arched surface faces the wave direction. (c) The voltage of various capacitors charging by the B-TENG. (d) The charging and discharging processes of a capacitor of 470 μF by the B-TENG under the water waves. (e) Photograph of an electronic thermometer driven by the B-TENG device under the water wave motions through charging a capacitor of 470 μF. (f) Photograph of dozens of LEDs, which are lit up by the B-TENG device under the water wave motions. Used with permission from Ref. [167]. Copyright (2018) Wiley-VCH.

Traditionally, the electromagnetic power generators for harnessing water wave energy are hampered by expensive fabrication costs, huge designs and poor energy conversion efficiencies at low frequencies [165]. Conversely, triboelectric nanogenerators (TENG) can harness energy from surroundings and exhibit several advantages including easy fabrication, high reliability and efficiency, large power outputs and low cost [166]. Recently, a butterfly-inspired triboelectric nanogenerator (B-TENG) was fabricated to harness water wave energy (Fig. 9) [167]. The B-TENG fitted with spring-supported four-bar linkage had different types of motions to capture multidirectional water wave energy. The springs stored the mechanical-trigger-generated potential energy and converted low-frequency motions into high-frequency oscillations, enhancing low-frequency waves harvesting capacity [168]. Outer arc design absorbed wave impact force and generated short-circuit current (75.35 μA), open-circuit voltage (707.01 V) and a maximum output power density (9.559 W m^−3^). This is enough energy to light up 180 light-emitting diodes. The generated power can be stored in a capacitor for marine sensor device operations. Therefore, B-TENG has potential in ocean information monitoring, energy generation and supply to ocean-facing locations and islands.

Clearly, abundant renewable energy resources and poor harnessing efficiencies in traditional technologies compel current methods to aim for high energy conversion efficiencies and improved performance [162]. Incorporation of bio-inspired systems aims to achieve high energy conversion efficiency, flexibility of devices, increased surface area and enhanced energy-harvesting capacity [163,168]. For example, the spider-silk-inspired dielectric nanocomposite exhibited a 200% improvement in discharged energy density at 150°C [104]. Such outstanding results propel current research to design new-age energy-harvesting technologies employing contemporary, sustainable and consistent bio-inspired principles. Thus, resultant bio-inspired renewable energy-harvesting technologies tap into the advantages of natural species and exhibit high energy-harnessing performance.

### Energy storage applications

Energy storage systems operate by storage of ions within two electrodes, coupled with an electron flow in an external circuit [169]. Effective systems demand enough ions to be delivered efficiently to electrodes, adequate electron amount in the external circuit, high energy density (store large quantity of energy) and high power density (charge and discharge quickly) [170]. To date, four types of energy storage devices exist, namely capacitors, supercapacitors, batteries and fuel cells. Comparatively, capacitors and supercapacitors have low energy density, while batteries and fuel cells have low power densities. Fuel cells have ultrahigh energy densities and require expensive precious metal catalysts [169,171]. With supercapacitors and batteries dominating the market, improving their energy and power densities ensures increased efficiency.

Efficient energy storage materials require high specific surface area and excellent connectivity [149]. Butterfly wings have high surface area 3D hierarchical microarchitectures for shortened transport and ion-diffusion pathways. The wings can be carbonized directly to form N-doped carbon with numerous active sites [88]. Fabrication of bio-inspired electrodes with transition metal oxide ensures improved capacity, ecological friendliness and a cheap product [172]. Together, the parameters produce high-performance electrodes for energy storage applications.

**Figure 10.**
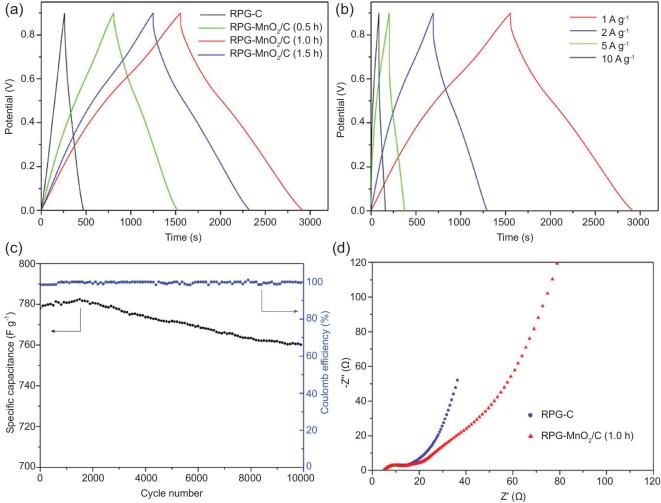
MnO_2_/C composite with a 3D hierarchical ridge/straight-pore/nano-grid architecture (RPG-MnO_2_/C) for supercapacitor applications. (a) Galvanostatic charge–discharge curves of RPG-C and RPG-MnO_2_/C with different MnO_2_ loadings at 1 A g^−1^. (b) Galvanostatic charge–discharge curves at different current densities for RPG-MnO_2_/C (1.0 h). (c) Cycling stability and coulomb efficiency of RPG-MnO_2_/C (1.0 h) at 10 A g^−1^. (d) Electrochemical impedance spectra of RPG-C and RPG-MnO_2_/C (1.0 h). Reprinted from Ref. [174], Copyright (2018), with permission from Elsevier.

Essentially, 3D grid architecture provides more active sites for electrodes and solves tangling by interconnected units in 1D architectures. On the contrary, 1D array architectures have effective electron/mass relay channels and high available surface area [173]. Hence, the green part of *Parides sesostris* butterfly wings contain subtle ridge/straight-pore/nano-grid architectures (with the combined 1D arrays and 3D grids). This inspired fabrication of MnO_2_/C composite with 3D hierarchical ridge/straight-pore/nano-grid architecture (RPG-MnO_2_/C), using a carbonizing surface reaction process for a supercapacitor electrode (Fig. 10) [174]. Maximum specific capacitance of 1539.7 F g^−1^ at 1 A g^−1^ and 97.6% capacitance retention at 10 A g^−1^ after 10 000 charge–discharge cycles, with ∼100% coulomb efficiency was recorded. The electrode performance stems from the combined effect of the composite (MnO_2_/C) and hierarchical 3D architecture.

Likewise, 3D hierarchical carbon-based architectures exhibit advantages including creation of hierarchical porous channels, high electrical conductivity and sustained superior structural mechanical stability [175]. Previously, *Morpho* wing inspired N-doped carbonized wing scale-cobalt oxide (CWs-Co_3_O_4_) nanopillar arrays were fabricated for supercapacitor applications [172]. The CWs-Co_3_O_4_ architecture exhibited a maximum specific capacity of 978.9 F g^−1^ at 0.5 A g^−1^, 94.5% capacitance retention over 2000 cycles and a maximum energy density increase to 99.11 Wh kg^−1^, without compromising the power density. Therefore, the carbon-based energy storage materials demonstrate good energy and power densities for energy storage applications.

Undoubtedly, electrode architecture in energy storage applications should exhibit high surface area and good light-trapping properties [170]. For instance, a leaf-inspired supercapacitor having leaves-on-branchlet architecture exhibited high areal capacitance of 2.35 F cm^−2^ and ∼95% capacitance retention over 10 000 cycles [176]. Similarly, plant architectures as bio-templates inspired design of electronic plant supercapacitors [177]. Therefore, bio-inspired energy storage systems show advantages including flexibility, stability, improved output power and energy densities, high surface area and improved light-trapping ability.

Overall, butterfly wing architectures have high specific surface area and light-trapping mechanisms for high-efficiency light absorption over a wide spectrum [148]. High specific surface offers active sites, light trapping of low-energy photons and shortened transport and ion-diffusion pathways. Additionally, a bio-inspired triboelectric nanogenerator showed improved water wave energy-harnessing capacity. Despite research outcomes reporting improved energy-harnessing efficiencies, the next generation of high-technology energy-harnessing systems should aim at mimicking high efficiencies in natural species. Improving conversion efficiencies of future bio-inspired energy-harnessing technologies will bridge the global energy deficit.

## SUMMARY AND FUTURE PROSPECTS

In this review, butterfly wing scale as the model biological system, has been classified according to the specialized regions. Our updated research progress highlighted the structural appearance of butterfly wing architectures, their distinctions based on modification of special structural elements in their microarchitecture and the application of butterfly wing properties in selected bio-inspired sensor and energy systems. Certainly, butterfly wing micro/nanoarchitecture replication promotes the improvement of physiochemical characteristics, light manipulation, selectivity, stimuli responsiveness, stability, biocompatibility and surface area [148]. As a growing field, butterfly wing architecture research has shown a competitive potential in comparison to similar sensor and energy applications inspired by other biological species [113,178]. For example, a report by Mwenze *et al*. used *Papillon junonia* butterfly to detect gasoline adulteration to as low as 4.7% adulteration [179]. Similarly, Davis *et al*. used finite-difference time-domain modeling to demonstrate that the ultra-black in some butterfly wings is due to expanded trabeculae and ridges, which increases absorption and reduces surface reflectance by 16-fold [180]. This recent butterfly wing architecture research progress and performance enhancement should propel future research to broaden the application scope to infrared detection and photothermal materials for stealth technologies, drug delivery and cancer imaging and therapy. In addition, the high surface area butterfly wing hierarchical architectures should inspire multipurpose photo-responsive materials. The current available knowledge provides an adequate platform to guide pioneering research in developing the next generation of bio-inspired functional materials with high sensing and energy-harnessing capabilities.

## Supplementary Material

nwaa107_Supplemental_File
