# Supplementary material for: Butterfly wing architectures inspire sensor and energy applications
Source: Natl Sci Rev. 2020 May 23;8(3):nwaa107. doi: 10.1093/nsr/nwaa107 (PMC8288439; doi:10.1093/nsr/nwaa107)
Supplement: nwaa107_Supplemental_File [file nwaa107_supplemental_file.docx]

**Butterfly wing architectures inspire sensor and energy applications**

Supporting Information

**Butterfly wing-inspired Fabrication**

Fabrication of butterfly wing inspired systems can be done according to any of three principles namely, modification, coating and filing or replication [1]. These principles are a generalized methodology that entails several fabrication techniques as shown in the summarized scheme (Fig S1). In discussing the principles, we highlight the process flow and explore selected fabrication techniques under each principle. In details, modification involves altering the architecture of the wing scale or incorporation of functional groups on wing templates. This causes a change in the initial characteristics of the butterfly wing template and has been applied in infrared (IR) sensors [2]. Conversely, coating entails bond creation between an applied layer of substrate (polymer or composite) and a butterfly wing template. The applied layer conceals the details of the wing template architecture. The product characteristics majorly rely on the thickness of the added layers. After coating, the detailed structure of the wing template cannot be maintained. Coating has been applied in fabrication of temperature, pH and gas/ethanol/vapor sensors [3-5].

Filling or replication principle entails attachment of components onto the butterfly wing template without concealing the original hierarchical architecture. Here, the original structure of the butterfly wing template is maintained. Filling has been applied in the fabrication of optical photonic devices, surface-enhanced Raman scattering (SERS) sensors and magnetic sensors [6]. While filling and coating add components to the wing template, their main difference is in the covering of the architecture. With various examples categorized under each principle, the fabricated systems may often vary in the synthesis technique and principle according to the requirements of the product [7]. A detailed discussion of each fabrication principle is highlighted below.


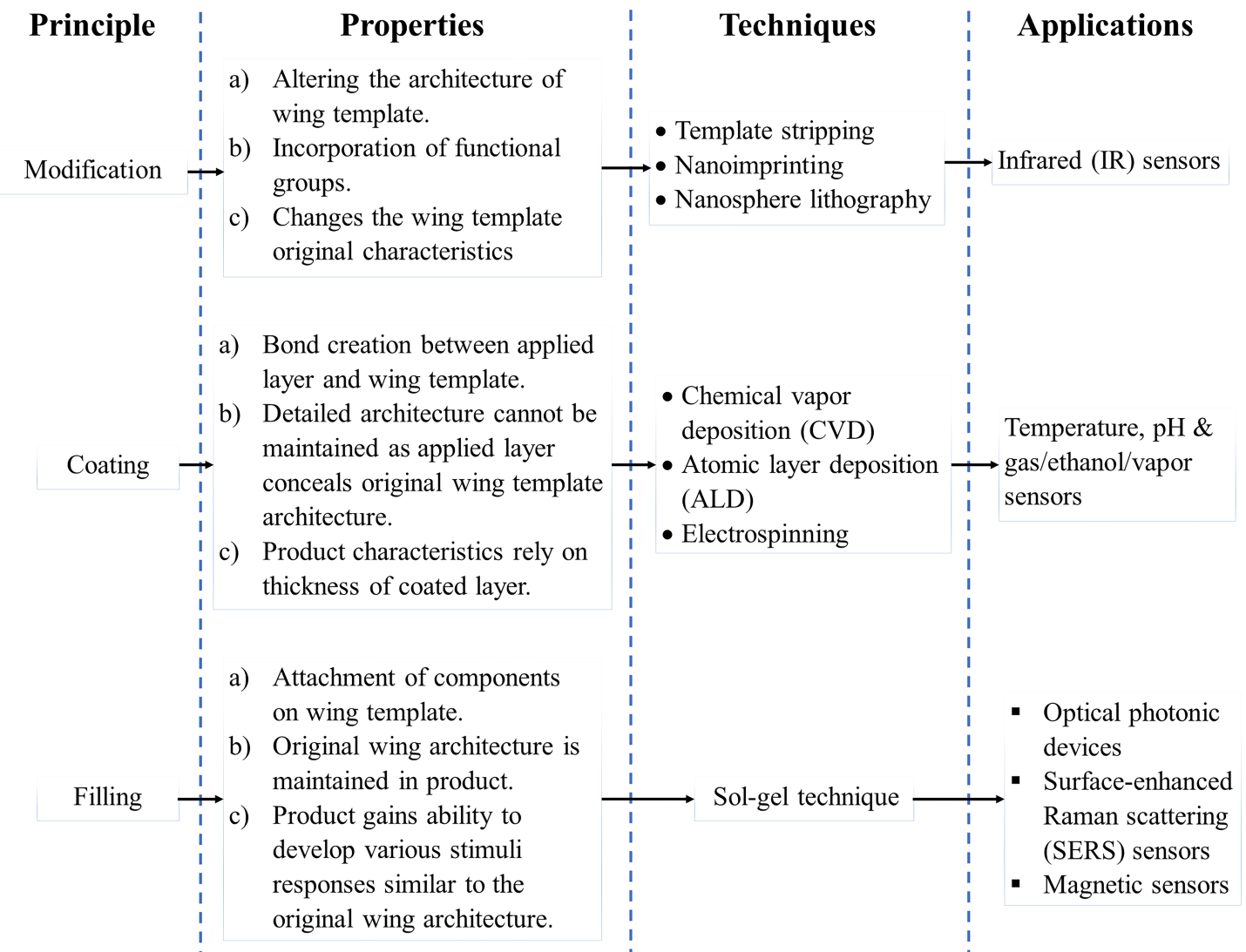


Figure S1. Scheme detailing the three fabrication principles.

**Modification**

This principle of altering the wing scale architecture entails additive or subtractive techniques such as template stripping, nanoimprinting lithography and nanosphere lithography. Template stripping involves creation of nonplanar metallic structures capable of tuning their optical properties by combining metal nanostructures with stretchable substrates [8]. Conversely, nanoimprinting lithography is a high-throughput methodology in lithography where a mold is pressed against a thermoplastic (polymer material), which has been heated above the glass transition temperature to fabricate patterned systems. The applied pressure makes the substrate conform to the intended pattern of the mold [9]. Nanoimprinting lithography has numerous advantages including high output capacity, ability to imprint large areas and low cost [10]. Another technique using modification principle is nanosphere lithography, which uses submicroscopic colloidal particles arranged in close packed arrays as masks on a template surface. Subsequently, the surface is exposed to an ion beam, light or vacuum deposition, followed by a lift-off process to remove the particles [11]. Ion beam etching results in isolated posts of template material while vacuum deposition yields holed thin films.

A previous report explored replication of *Morpho peleides* upper laminae by imprinting of the upper-wing surfaces to produce a positive epoxy resin imprint [12]. The process of fabrication included: (i) Mounting *Morpho peleides* wing on a microscope slide with the blue surface facing upwards. The mounted specimen is then placed inside a reservoir. (ii) Pioloform (PF) solution formed by dissolving PF powder in chloroform is filled into the reservoir and drained off at 1.2 mL s^-1^ velocity. The chloroform is evaporated at ~10^o^ angle, resulting in wing coated with PF. (iii) Polyvinylsiloxane (PVS) applied on mounted wing specimen. Then, various wing pieces upper laminae touched once on the smooth PVS layer for 10 min to create negative imprints. (iv) The formed positive upper-wing imprint produces a negative cast (master stamp) when organic material is removed by washing in chlorine and distilled water baths. (v) PVS master stamp is rested at a horizontal position on an air-bubble-free epoxy resin glass slide mounting for at least 5 min. The PVS master stamp is then removed, leaving behind a positive epoxy resin imprint. As a result, the PVS master stamp developed many positive replicas of up to 3 cm^2^ in an hour, having visible structural coloring effects.

Similarly, nanosphere lithography and commercial DVD nanoimprinting techniques recently used *Papilio paris* butterfly architectures to fabricate a perovskite photodetector with a light trapping hierarchical architecture (1D nano-grating bonded porous 2D photonic crystal perovskite photodetector, G-PCPD) [13]. The nanoimprinting process entailed four steps. (i) An air-liquid self-assembly method joined a template of 2D crystalline colloidal arrays onto a glass substrate. (ii) Spin-coating of the perovskite precursor onto 2D crystalline colloidal arrays via an antisolvent technique to prepare a perovskite film. (iii) Development of grating architecture by commercial DVD nanoimprinting technique. (iv) Evaporation of an Au electrode on the nanoimprinted film. The G-PCPD architecture allowed a rate of -0.72 nA/º in polarized-sensitive light detection. Similarly, the white light detection and response of the G-PCPD rose to 12.67 A/W and 6.28×10^13^ Jones, almost 8 times more than in pristine photodetector.

**Coating**

Coating principle involves bonding of applied polymer or composite with an underlying template surface to form a film. The coated film covers details of the underlying architectures. Also, the film properties majorly relying on the thickness of the added coating layers [1]. A common method used to replicate bio-templates using coating principle is the vapor phase deposition (VPD) process that transforms gas phase to solid phase in mostly thin-film coating [14]. Two major VPD techniques namely, chemical vapor deposition (CVD) and atomic layer deposition (ALD) rely on successive use of gas phase chemical method. Importantly, CVD is a chemical process that transforms gaseous molecules into solid coat materials on the surface of a substrate or template [15]. Despite the similarity in the two techniques, ALD reaction breaks CVD reaction process into twin half-reactions and maintains separation of the precursor materials through the reactions.

A recent report fabricated a structurally colored water purification photocatalyst by replicating *Morpho sulkowskyi* architectures via deposition of nanocrystalline ZnO coatings on the butterfly wings using low temperature ALD [16]. ALD was selected due to self-limiting surface reactions, resulting in depositing of conformal thin-films over 3D surfaces. In addition, it maintains the structural integrity of the underlying wing template. The ALD ZnO films were deposited at low temperature (<150^o^C) on *Morpho sulkowskyi* wings to ensure limitation of post annealing high temperature processes that would damage the wing template. The ZnO coatings applied by ALD can adjust the photocatalyst structural coloration across the visible spectrum. Additionally, they exhibit optimal coat thickness that maximizes photoactivity. The high catalytic activity is ascribed to the balance between absorption of light and quantum yield with increment in coating thickness.

Electrospinning is a coating technique used to fabricate uninterrupted fibers at submicron to nanoscale size range [17]. Typical electrospinning systems comprise of a high-power supply, conducting collector, a syringe pump and high molecular weight polymer. To commence electrospinning process, an electric potential is applied between the conducting collector and syringe nozzle, to create a high electric field. The polymer solution is extruded from the distorted droplet at the tip of the syringe nozzle to form a charged solution jet, which is deposited on the conducting collector. The charged jet has enough time to completely evaporate the solvent and form a solid fiber membrane during deposition on the collector [18]. Recently, replication of phototunable *Morpho* butterfly architectures was achieved by electrospinning through deposition of azobenzene-containing linear liquid crystal polymer (LLCP) on the wing template. The resultant 3D bi-layered microarchitectures hierarchically deformed on UV light irradiation, causing a reflection peak blueshift (70 nm) and a marked change on reflectance (40%). These results present a potential opportunity for the application of electrospun bio-architectures in cosmetics, pigments and sensors.

**Filling or Replication**

This principle entails addition of components to mimic or replicate the actual hierarchical structure without covering the original hierarchical architecture, while attaining the ability to develop various stimuli responses [1]. *Sol-gel* technique is the most common filling wet method in the fabrication of butterfly wing inspired systems. Sol-gel technique is simple, fast, inexpensive and flexible. It generally entails a chemical conversion by assembly of functional materials, followed by removal of the template [19]. The sol-gel technique consists of three basic steps [20]: (i) Pretreating the butterfly wing template by immersion into dilute acid, ethanol solution or deionized water. Then, washing with deionized water to eliminate the residue on the surface and drying in air. (ii) Functionalizing the chitinous surface by immersion into a functionalization solution (usually ethane diamine ethanol solution), to insert functional groups such as amino groups. (iii) Deposition of the metals or semiconductors onto the template surface by immersing the functionalized wing sample into precursor solutions of the metals or semiconductors. In addition, calcination of as-prepared specimen may be done in vacuum environment or in air to maintain or eliminate the wing template, respectively.

Recently, a research report inspired by butterfly, cicada and dragonfly wings as bio-templates, used *sol-gel* method in fabricating electrocatalysts for oxygen reduction reactions in metal-air batteries and fuel cells [21]. The synthesis strategy to fabricate butterfly wing inspired graphene-graphite films (GGFs) entailed pretreatment of the butterfly wings and heating the wing coated with FeCl_3_ in N_2_ environment. This ensured the optimal utilization of the natural organism characteristics. In this methodology, the wings were a source of GGF generation and a porous supportive architecture for doping with nitrogen. They also ensured 2D spreading and two-face exposure of the GGFs. The GFFs had 0.942 V half-wave potential and <0.07% H_2_O_2_ yield in electrocatalysis. These results are comparable to previous oxygen reduction reactions catalysts in alkaline conditions and those of the best commercial Pt/C.

Another research report used *sol-gel* synthesis technique to fabricate gyroid-structured TiO_2_ photonic crystals (GTPCs) using *Callophrys* rubi butterfly wings as the soft bio-template [22]. The synthesis process entailed pretreatment in ethanol, amination in dilute nitric acid, deposition of TiO_2_ from precursor solution, heat treatment and sintering. The research reported novel control of the volume fraction (VF) through regulation of rinsing time of the wing templates in the precursor solution. The resultant GTPCs had different volume fractions of 3.2%, 8.0% and 13.5%. The results pave way for further exploitation of GTPCs optical performance in visible light spectrum, which surpasses previous simulation results.

In summary, preference of the fabrication principle and technique depends on the requirements in butterfly wing inspired product. In certain systems, the combination of filling and modification or coating can produce novel effects. Filling is a process that is easy to execute and is commonly applied in systems with difficulty in control of product morphology and where strict accuracy is not mandatory. Conversely, modification and coating are characterized by chemical bonding. They maintain accurate control of product morphology through regulation of etching duration or by regulation of the coating layer thickness. Undoubtedly, there are numerous fabrication techniques which apply the principles above in the design of bio-inspired sensor and energy materials. Herein, we have sampled some of them to explain the principles utilized in fabrication. These numerous techniques are propelled by research aimed at achieving higher efficiency and performance in the product. Therefore, a great potential exists for the improvement of bio-inspired fabrication, especially in sensors and energy applications.

**Reference**

1. Zhu Y, Zhang W and Zhang D. Fabrication of Sensor Materials Inspired by Butterfly Wings. *Adv Mater Technol* 2017; **2**(7): 1600209.

2. Rogalski A. History of infrared detectors. *Opto-Electron Rev* 2012; **20**(3): 279-308.

3. Lu T, Zhu S, Ma J *et al.* Bioinspired Thermoresponsive Photonic Polymers with Hierarchical Structures and Their Unique Properties. *Macromol Rapid Commun* 2015; **36**(19): 1722-8.

4. Yang Q, Zhu S, Peng W *et al.* Bioinspired fabrication of hierarchically structured, pH-tunable photonic crystals with unique transition. *ACS Nano* 2013; **7**(6): 4911-8.

5. Wang LL, Jackman JA, Park JH *et al.* A Flexible, Ultra-sensitive Chemical Sensor with 3D Biomimetic Templating for Diabetes-Related Acetone Detection. *J Mat Chem B* 2017; **5**(22): 4019-24.

6. Tan Y, Gu J, Xu L *et al.* Biological Templates: High-Density Hotspots Engineered by Naturally Piled-Up Subwavelength Structures in Three-Dimensional Copper Butterfly Wing Scales for Surface-Enhanced Raman Scattering Detection. *Adv Funct Mater* 2012; **22**(8): 1542-42.

7. Potyrailo RA, Karker N, Carpenter MA *et al.* Multivariable bio-inspired photonic sensors for non-condensable gases. *J Opt* 2018; **20**(2).

8. Zhang W, Gu J, Liu Q *et al.* Butterfly effects: novel functional materials inspired from the wings scales. *Phys Chem Chem Phys* 2014; **16**(37): 19767-80.

9. Amsden JJ, Domachuk P, Gopinath A *et al.* Rapid nanoimprinting of silk fibroin films for biophotonic applictions. *Adv Mater* 2010; **22**: 1746.

10. Hirai Y, Harada S, Isaka S *et al.* Nano-Imprint Lithography Using Replicated Mold by Ni Electroforming. *Jpn J Appl Phys* 2002; **41**(Part 1, No. 6B): 4186-89.

11. Burmeister F, Schafle C, Keilhofer B *et al.* From mesoscopic to nanoscopic surface structures: Lithography with colloid monolayers. *Adv Mater* 1998; **10**(6): 495.

12. Zobl S, Salvenmoser W, Schwerte T *et al.* Morpho peleidesbutterfly wing imprints as structural colour stamp. *Bioinspir Biomim* 2016; **11**(1): 016006.

13. Zhan Y, Wang Y, Cheng Q *et al.* A Butterfly-Inspired Hierarchical Light-Trapping Structure towards a High-Performance Polarization-Sensitive Perovskite Photodetector. *Angew Chem Int Ed Engl* 2019; **58**(46): 16456-62.

14. Zhang D, Zhang W, Gu J *et al.* Inspiration from butterfly and moth wing scales: Characterization, modeling, and fabrication. *Prog Mater Sci* 2015; **68**: 67-96.

15. Tiwari JN, Tiwari RN and Kim KS. Zero-dimensional, one-dimensional, two-dimensional and three-dimensional nanostructured materials for advanced electrochemical energy devices. *Prog Mater Sci* 2012; **57**(4): 724-803.

16. Rodriguez RE, Agarwal SP, An S *et al.* Biotemplated Morpho Butterfly Wings for Tunable Structurally Colored Photocatalysts. *ACS Appl Mater Interfaces* 2018; **10**(5): 4614-21.

17. Sun JY and Bhushan B. Nanomanufacturing of bioinspired surfaces. *Tribol Int* 2019; **129**: 67-74.

18. Liu H, Wang Y, Huang J *et al.* Bioinspired Surfaces with Superamphiphobic Properties: Concepts, Synthesis, and Applications. *Adv Funct Mater* 2018; **28**(19): 1707415.

19. Lu T, Peng W, Zhu S *et al.* Bio-inspired fabrication of stimuli-responsive photonic crystals with hierarchical structures and their applications. *Nanotechnology* 2016; **27**(12): 122001.

20. Zhang MF, Meng JT, Wang DP *et al.* Biomimetic synthesis of hierarchical 3D Ag butterfly wing scale arrays/graphene composites as ultrasensitive SERS substrates for efficient trace chemical detection. *J Mater Chem C* 2018; **6**(8): 1933-43.

21. Li HY, Zhang LH, Li L *et al.* Two-in-one solution using insect wings to produce graphene-graphite films for efficient electrocatalysis. *Nano Res* 2019; **12**(1): 33-39.

22. Wu LP, Wang WL, Zhang W *et al.* Optical Performance Study of Gyroid-Structured TiO_2_ Photonic Crystals Replicated from Natural Templates Using a Sol-Gel Method. *Adv Opt Mater* 2018; **6**(21): 8.
